# Family-Focused Public Health: Supporting Homes and Families in Policy and Practice

**DOI:** 10.3389/fpubh.2019.00059

**Published:** 2019-03-20

**Authors:** Carl L. Hanson, Ali Crandall, Michael D. Barnes, Brianna Magnusson, M. Lelinneth B. Novilla, Jaron King

**Affiliations:** Department of Public Health, Brigham Young University, Provo, UT, United States

**Keywords:** family health, family focused interventions, public health 3.0, life expectancy, public health policies

## Abstract

Life expectancy in the US is on the decline. Mental health issues associated with opioid abuse and suicide have been implicated for this decline necessitating new approaches and procedures. While *Public Health 3.0* provides a call to action for stakeholders to work closely together to address such complex problems as these, less attention has been given to engaging and supporting the most important stakeholders and primary producers of health within the US: families and households. The idea that health begins at home is discussed from the perspective of primary, secondary, and tertiary prevention levels. Primary prevention where research provides evidence for the role of the family in healthy child development. Secondary and tertiary prevention where research offers evidence for the role of the family in caregiving. Despite this evidence, greater focus and attention must be placed on the family at all prevention levels as an often overlooked setting of public health practice and level of influence. Prevention across all levels is enhanced as public health practitioners *think family* when designing and implementing public health policy. Four family impact principles are presented to help guide planning and implementation decisions to nourish family engagement.

For the second year in a row, life expectancy in the United States has declined (1). While age-adjusted death rates have decreased for seven of the leading causes of death, rates have increased for unintentional injuries and suicide (1) Behavioral health challenges such as opioid abuse and suicide have been implicated for these increases (2, 3). Addressing the causal factors for these conditions with macro-level interventions is a public health imperative and responsibility. However, current approaches are not bending the curve as the prevalence of chronic conditions and mental illness remain high. Besides, they account for 86% of the $2.7 trillion annual healthcare expenses in the US (4). Successfully tackling twenty -first century public health challenges—such as the leading causes of death—requires greater nurturing and support of the system through which health is most directly produced: the family.

The integration of ecosystems within models of health has helped to emphasize their importance for well-being, public health, and disease prevention (5). Newer models tend to be more expansive in nature and incorporate the impact of a global ecosystem, built and natural environments, government and policy, local economies, and the community (5). Nested deep within these systems models is the individual whose growth and development is primarily influenced by interactions with friends, social networks, and most notably the family.

Viewing health from an ecological perspective certainly draws attention to the powerful influences of environmental determinants. The importance of organizational partnerships and collaboration for health was emphasized in the 2016 U.S. Department of Health and Human Services (HHS) Call to Action entitled *Public Health 3.0* (6). *Public Health 3.0* outlines a twenty -first century approach to American population health and prevention and emphatically encourages public health departments to engage with community stakeholders, and thus embraces ecosystems as a necessary component of health and ultimately prevention. While the role and responsibilities of organizations within the public health system must involve strong leadership, partnerships, funding, relevant data, and a foundational infrastructure as encouraged by *Public Health 3.0*, addressing public health challenges may best be achieved as practitioners consider the influence of policies and practices on families and households. Indeed, though other settings of practice were also excluded from Public Health 3.0, families are likely among the most important stakeholders to create a twenty -first century public health infrastructure *Public Health 3.0*. While the nation's leading health promotion and disease prevention initiative *Healthy People 2020* has adopted the ecological goal to “create social and physical environments that promote good health for all,”(7) objectives and measures remain focused on individual health. Through this paper, we reemphasize the vital role of family health and provide guiding principles as to how practitioners can better *think family* as policies are developed and implemented.

## Public Health Begins at Home

In recent history, the term “family health” has been used to represent anything from maternal and child health to reproductive health. Rarely has family health encompassed the family as an essential context for the development of health, including all family members across time and setting. One major stride toward the inclusion of the family as an important entity of health comes from the Center for Disease Control and Prevention (CDC) site on the family, which coincidentally, is managed by the Center's office of women's health (see https://www.cdc.gov/family/). We propose that the family should be considered as the basic unit of health production at the individual and societal level, a context in public health practice, and an essential part of public health policy, research, and teaching.

Several models have been used to illustrate the important connection between families and health in general. These models mostly agree that health is socially constructed within the family who resides in a household that is embedded in larger contextual systems such as the community and society (8–10). Family values, behaviors, routines, and decisions, borne out of the recurring patterns of interactions within and outside the home have significant implications on the health of other members in the household (11, 12). The Family Health Model provides an ecological perspective where the production of health is based on contextual, functional, and structural domains (10). Research has demonstrated the influence of contextual and functional aspects of long-term health and well-being across the life course (12). The idea that health begins in the home and is influenced by the family is not only supported by theoretical models but also scientific evidence and family-focused interventions at the primary, secondary and tertiary prevention levels.

### Primary Prevention

The goal of primary prevention efforts is to prevent disease or injury from occurring. While the role of the family in primary prevention is important across the life-course, the lack of primary prevention efforts that involve the family of children and adolescents can lead to adverse health outcomes later in life (13). Findings from CDC-Kaiser Permanente Adverse Childhood Experiences (ACEs) study revealed that exposure to childhood emotional, physical, or sexual abuse, and household dysfunction was related to increased risk for numerous negative health outcomes correlated with a lower life expectancy. These health outcomes included but were not limited to alcoholism and alcohol abuse; chronic obstructive pulmonary disease; depression; illicit drug use; ischemic heart disease; intimate partner violence; smoking; suicide attempts; early initiation of sexual activity; sexual violence; and lower academic achievement. Psychological processes maintained by individuals are influenced by these contexts which include social interactions within families/households and are experienced across the life course. While these findings suggest family protection in childhood and adolescence is extremely important, family protection in young, middle, and older adulthood should be no less important. For example, strain in marriage and cohabiting relationships have been implicated in partner physical and mental illness and higher mortality rates (14), whereas the absence of connections with family (such as living alone or otherwise experiencing feelings of loneliness) may also result in increased mortality (15).

**Figure 1:**
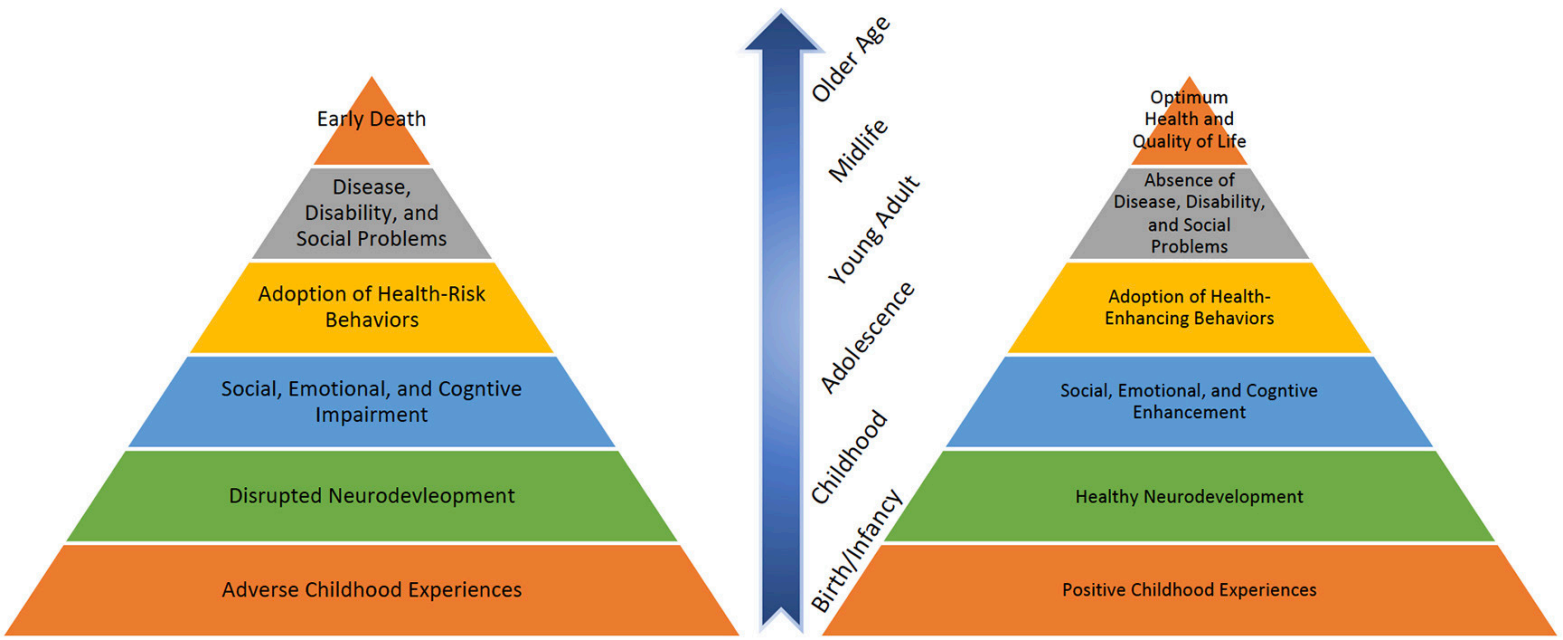
Mechanism through which childhood experiences influence health outcomes.

While research has revealed the impact of adverse childhood experiences on health and well-being across the life-course, other findings have shown the effect of positive experiences, primarily through the family. Studies indicate that families can produce positive health outcomes through optimal youth and child development by building protective factors such as parental resilience, social connections, knowledge of parenting and child development, concrete support in the times of need, and social and emotional competence in children (16). Figure 1 displays the life-course impact of positive as well as adverse family experiences on health.

Many clinical settings in public health have wholeheartedly embraced the importance of the family already as it relates to healthy child development and primary prevention. The Nurse-Family Partnership is on primary prevention strategy that provides lower-income families with home visits from registered nurses. Visits have helped to improve maternal and child health as well as economic security (17). From mental health services with their vast discipline of family psychology to medicine with family health history as a diagnostic tool. Family history has been utilized to aid practitioners in treatment decisions but also helps family members as they look to navigate the complexity of the American healthcare systems. As the role of genetics plays an increasingly significant role in health maintenance, public health practitioners can assist families best by understanding how a family has produced health through multiple generations, if at all possible.

Other public health primary prevention bright spots where families are recognized as important producers of health include the Communities That Care (CTC) system (18) and asthma home visiting programs. The CTC system utilizes community-based coalitions to identify needs and implement programming to address adolescent risk and protective factors associated with violence, substance abuse, teen pregnancy, school dropout, and delinquency. While efforts to address these social morbidities are rooted in individual/peer, family, community, and school domains, recent evidence suggests that the low saturation or reach of CTC programming among families has contributed to no evidence of protection in this important domain area (19). Asthma home visiting programs place a focus on the family by bringing public health practitioners into the homes of families with cases of childhood asthma. These programs allow practitioners to address environmental factors with entire families in real-time, and often exist as an extension of hospital visits (20).

### Secondary and Tertiary Prevention

Health production in the home may not be limited to primary prevention activities alone. The goal of secondary prevention is early diagnosis and treatment while tertiary is rehabilitation. These efforts can encourage maintaining and/or adopting household health practices that are vital in addressing existing chronic conditions such as heart disease, cancer, diabetes, and disability, including the changes associated with aging. These conditions can “occur and cycle in flare-ups” across the life-course (21). In such situations, the family, such as the spouse/partner and older children, functions primarily as an informal caregiver, providing unpaid ongoing assistance. Relatives, friends, and the family's other social support network, such as neighbors and church, can step into this role as a source of additional support (22).

The vital role of the family in assisting individuals with chronic conditions has been well-established, and several secondary and tertiary prevention models designed to increase family support have been identified (23). These models include strategies to (1) guide family members in goal setting activities related to supporting patients, (2) teach family members supportive communication techniques that encourage patients, and (3) teach family members how to monitor symptoms and treatments associated with chronic conditions (23). A more recent review of healthcare interventions among adults with chronic conditions revealed that combining family-centered approaches with active learning strategies, transitional care, and follow-up were instrumental for achieving positive patient health outcomes (24).

For older adults with chronic or disabling conditions, the American Association for Retired Persons (AARP) has led out with a policy statement related to person-and family-centered care (25). Through this statement, AARP emphasizes the important roles of family caregivers which include but are not limited to: (1) providing daily help for thus with functional limitations, (2) negotiating with healthcare and social service professionals, (3) coordinating care and supportive services, and (4) managing continuity of care. Because of these critical roles, there is a concern for family caregivers and their need to receive information and support to function in their roles, manage stress due to social isolation, and balance the demands and strains associated with caregiving that can lead to burnout. Although traditionally expected to step into the caregiving role, the family can help plan ahead for the needs of older loved ones. For instance, aging in place requires financial, economic, and legal planning as early as during one's working years—way before the changes of aging occur. Beyond addressing the inevitable physical, mental, and emotional changes of aging through caregiving, the family plays a critical role in helping a loved one plan ahead to maintain independence, comfort, quality of life, and the ability live in the place of one's choice as for as long as one can with access to supplementary services (26).

Indeed, public health begins in the home where families reside (27). Its capacity to nurture, care, protect, teach and influence makes the family a practical entry point in the promotion and maintenance of individual and collective health (11).

## Supporting Families for Health Production

The ecological perspective nests the family within the broader domains of community programs, services, and organizations; the local economy; and government/policy. As such, ensuring that primary, secondary, and tertiary prevention efforts (nurturing services, programming, and policies) are offered through these larger domain areas is critically important to the health of the family and ultimately the individual. Failure to acknowledge the influence of the family as producers of health means an inability to develop and implement programs and policy that support and strengthen family functioning and produce conditions through which families can thrive.

If homes are where families reside and where health or illness is most fundamentally produced, public health efforts must expand approaches that have a life-course perspective in mind and shift greater attention to supporting families and households. Because settings of practice for public health professionals often include individuals within communities, schools/universities, health care, and business/industry, the very nature of these settings may make it difficult for practitioners to remember the role of family and households as well as reach them with programming or services. Public health practitioners who make this shift learn to *think family* (10) as they assess, plan, and implement programming across contextual, functional, structural domains.

### Thinking Family

*Thinking family* can be done as public health practitioners carefully consider in their practice several family impact principles (28). We have adopted four guiding principles to a public health model. These principles, widely accepted by family science scholars, were initially developed to achieve the goal of developing programs and policies that support and strengthen families across the life-course and in diverse settings (29). As such, the principles are meant as discussion starters to help public health practitioners design new and improve existing programs to better support families produce positive health outcomes at the individual, family, and community level. However, the principles should be applied based on the context of each community and priority population. The adaptations to the principles were made to be specific to public health programming efforts, and are aligned with population-based prevention efforts. These principles include family engagement, family responsibility, family stability, and family diversity.

### Family Engagement

Family engagement requires that practitioners *think family* by establishing strong partnerships between their programming and families, including involving families as stakeholders in the planning, implementation, and evaluation of programs. As noted earlier, each family represents a unique dynamic of everything from communication to routines., programming must respect the autonomy and culture of involved families. Successful family-focused programs in public health might: (1) consider how they can include families as key stakeholders in development and implementation, (2) train staff to respect family decision and choices, (3) examine how to provide services or programming to the entire family unit (e.g., beyond mother and child), and (4) help connect families with needed community resources related to health issue(s) of concern. Aligned with community participatory action research, models exist for involving families as ongoing advisors, co-evaluators, and even leaders in programs and evaluation efforts (30).

### Family Responsibility

Family responsibility requires that practitioners *think family* by planning and delivering programming that supports and empowers families in performing traditional functions that they should complete across diverse family structures. Examples of these functions may include family formation, partner relationships, economic support, child rearing, and caregiving. Practitioners who *think family* also support the family's choices in how they perform these functions. Categorical programs that focus on a specific disease or determinant in public health might: (1) enable families to better fulfill their responsibilities as it pertains to health by ensuring they are helping families to build their capacity to develop the necessary knowledge and skills, and (2) avoid being overly taxing on family time and other resources by providing services at flexible times and locations and ensuring that policies to receive services are presented without bureaucratic barriers.

### Family Stability

Family stability requires that practitioners *think family* by planning and delivering programming that encourages balance within the family and recognizes the importance of family relationships to individual family functioning and health in the short-term and over time. Disease or determinant focused programs in public health might: (1) work to help families avoid health problems before they become serious and chronic, (2) help families maintain healthy routines in the face of change and stress related to health issue(s), (3) help families recognize that individual development, well-being, and behavior regarding the health issue(s) impact relationships within the family. Family stability may be particularly challenging in vulnerable populations that face challenging social and economic risk factors and who may be less likely to have positive role models (31). Strong social support has been associated with lower morbidity and mortality (32), and may be an important area of focus for practitioners working with populations that experience less economic and family stability.

### Family Diversity

Family diversity requires that practitioners *think family* by understanding interventions can have varied effects on different types of families and that family types are of increasing variety and importance in the twenty-first century (33). They acknowledge and respect the diversity of family life and do not discriminate against or penalize families based on cultural or ethnic background, economic situation, family structure, geographic locale, the presence of special needs, or religious affiliation. Categorical programs in public health might: (1) provide programming that is available and accessible to all family types based on culture, geography, and structure, and (2) ensure programming addresses the root causes of health issue(s) rather than symptoms. There are many good examples of programs that reach out to diverse family types that practitioners can model their intervention approaches after such as the Family Check-Up (34), and the Chicago Parent Program (35).

As social and environmental circumstances vary widely, the application of each principle should be adapted to the context of the community. For example, family responsibilities may vary based on culture and the diverse nature of families. Additionally, select principles at the time more require more attention and focus compared to others. For example, practitioners working with vulnerable populations face more social determinant risk factors. Such programs might initially center less on family responsibility while they work on engaging diverse families and linking them with healthy social supports.

## Conclusion

The decline of life expectancy in the US has prompted public health to search for new solutions. Theoretical models illustrate the critical role of the family, and family-focused primary, secondary and tertiary prevention strategies have yielded positive health outcomes. Whereas, health is ultimately produced in the home and within families, a greater focus on the family through public health interventions has the potential to create positive outcomes. These intervention outcomes may not only affect an individual and one's family at a time of need but through learned skills and interactions, the consequences may be built up or revised over the life-course. Given the importance of family/household on health and well-ness outcomes at the primary, secondary and tertiary prevention levels, practitioners can *think family* by giving greater attention to intervention strategies that help strengthen and support the family. Though the unique family and home configurations exist, several family impact principles can be applied to support each family's capacity for improved health production. While family-focused programming may seem more challenging at first, public health professionals should consider the support and strength offered by families to proactively improve health according to the capacity of each household unit. Public health researchers also have an important opportunity to increasingly recognizing the challenges of families as health producers and be prepared to incorporate many methodological approaches to more effectively *think family*. In response to HHS' 2016 Call to Action *Public Health 3.0* (6), families as important stakeholder may represent the missing link between healthcare and public health involvement. As the US public health infrastructure looks to reverse the decline of life expectancy, we call for a return to the home and the ultimate producer of health: the family.

## Author Contributions

All authors listed have made a substantial, direct and intellectual contribution to the work, and approved it for publication.

### Conflict of Interest Statement

The authors declare that the research was conducted in the absence of any commercial or financial relationships that could be construed as a potential conflict of interest.
